# Expression of genes involved in carbohydrate‐lipid metabolism in muscle and fat tissues in the initial stage of adult‐age obesity in fed and fasted mice

**DOI:** 10.14814/phy2.13445

**Published:** 2017-10-16

**Authors:** Nadezhda M. Bazhan, Alexandr V. Baklanov, Julia V. Piskunova, Antonina J. Kazantseva, Elena N. Makarova

**Affiliations:** ^1^ Laboratory of Physiological Genetics The Siberian Branch of the Russian Academy of Sciences The Federal Research Center Institute of Cytology and Genetics Novosibirsk Russia; ^2^ Department of Physiology Novosibirsk State University Novosibirsk Russia

**Keywords:** 16 h fasting, adult age obesity, C57Bl mice, carbohydrate‐lipid metabolism, WAT BAT muscle gene expressions

## Abstract

C57Bl mice exhibit impaired glucose metabolism by the late adult age under standard living conditions. The aim of this study was to evaluate white adipose tissue (WAT), brown adipose tissue (BAT), and skeletal muscle expression of genes involved in carbohydrate‐lipid metabolism at postpubertal stages preceding the late adult age in C57Bl mice. Muscle mRNA levels of uncoupling protein 3 (*Ucp3*) and carnitine palmitoyltransferase 1 (*Cpt1*) (indicators of FFA oxidation), WAT mRNA levels of hormone‐sensitive lipase (*Lipe*) and lipoprotein lipase (*Lpl*) (indicators of lipolysis and lipogenesis), muscle and WAT mRNA levels of the type 4 glucose transporter *Slc2a4* (indicators of insulin‐dependent glucose uptake), and BAT mRNA levels of uncoupling protein 1 (*Ucp1*) (indicator of thermogenesis) were measured in fed and 16 h‐fasted mice in three age groups: 10‐week‐old (young), 15‐week‐old (early adult), and 30‐week‐old (late adult). Weight gain from young to early adult age was not accompanied by changes in WAT and BAT indexes and biochemical blood parameters. Weight gain from early to late adult age was accompanied by increased WAT and BAT indexes and decreased glucose tolerance. Muscle *Ucp3* and *Cpt1* mRNA levels and WAT *Lipe* and *Slc2a4* mRNA levels increased from young to early adult age and then sharply decreased by the late adult age. Moreover, BAT *Ucp1* mRNA level decreased in the late adult age. Fasting failed to increase muscle Cpt1 mRNA levels in late adult mice. These transcriptional changes could contribute to impaired glucose metabolism and the onset of obesity in late adult mice during normal development.

## Introduction

In humans and rodents, the prevalence of obesity increases from birth until middle age (Barzilai et al. 1998; Facchini et al. 2001; Mizuno et al. 2004). In laboratory mice, middle age includes the period from 9 to 12 months (Flurkey et al. 2007; Jacobson 2002; Rusli et al. 2016). However, long before middle age in the late adult age (6 months) mice demonstrate impaired glucose metabolism in which insulin and blood glucose concentrations increase (Stenbit et al. 1997) and sensitivity to insulin decreases (Mizuno et al. 2004). The physiological mechanisms causing dysregulation of carbohydrate metabolism in the late adult age during normal development are unknown.

White and brown adipose tissues and skeletal muscle are known to be the main peripheral metabolic organs. It has been reported, that fasting regulates the transcription of many genes involved in carbohydrate‐lipid metabolism in these tissues in rodents (Camps et al. 1992; De Lange et al. 2006; Sánchez et al. 2009). One can assume that both the basal transcription and transcriptional response to fasting of these genes change with age. To understand the complex series of events that occur during postpubertal development, we examined age‐related changes in gene expression in metabolic organs (white and brown adipose tissues and skeletal muscles) in fed and fasted C57Bl mice at postpubertal stages preceding the middle age. Body weight, triacylglyceride (TG), free fatty acid (FFA), insulin and glucose plasma concentrations, and glucose blood concentrations by oral glucose tolerance test (OGTT) were considered as indicators of carbohydrate and lipid metabolism. The following parameters were measured in fed and 16 h‐fasted mice: muscle mRNA levels of uncoupling protein 3 (*Ucp3*) and carnitine palmitoyltransferase 1 (*Cpt1*) (indicators of FFA oxidation); WAT mRNA levels of hormone‐sensitive lipase (*Lipe*) and lipoprotein lipase (*Lpl*) (indicators of lipolysis and lipogenesis); muscle and WAT mRNA levels of the glucose transporter *Slc2a4* (indicator of insulin‐dependent glucose uptake); and BAT mRNA levels of uncoupling protein 1 (*Ucp1*)(indicator of thermogenesis).

This study shows that C57Bl mice during normal development have increased WAT and BAT indexes and decreased glucose tolerance at the late adult stage. Our findings suggest that these changes may be caused by age‐related decline in enzyme system activities for *β*‐oxidation of FFA in muscles, TG lipolysis in WAT, and thermogenesis in BAT. Our data provide new information on the mechanisms underlying obesity and dysregulation of carbohydrate‐lipid metabolism prior to middle age in mice.

## Methods

### Ethics approval

All experiments were performed in accordance with the “European Convention for the Protection of Vertebrate Animals used for Experimental and other Scientific Purposes” and the Russian national instructions for the care and use of laboratory animals. The protocols were approved by the Independent Ethics Committee of the Institute of Cytology and Genetics, Siberian Branch, Russian Academy of Sciences (protocol No 35 of October 26, 2016). All efforts were made to minimize animal suffering and reduce the number of animals used.

### Animals and experimental protocol

C57Bl mice were bred in the vivarium of the Institute of Cytology and Genetics (Siberian Branch, Russian Academy of Sciences, Novosibirsk). The mice were housed under a 12:12‐h light–dark regimen at an ambient temperature of 22°C. The mice were provided ad libitum access to commercial mouse chow (Assortiment Agro, Moscow region, Turacovo, Russia) and water. Male mice were separated from mothers at 28 days old and housed individually until treatment.

The animals were randomly divided into three age groups: 10 weeks (young mice), 15 weeks (early adult mice), and 30 weeks (late adult mice) according to the classification scheme by Flurkey et al. (2007). At the appropriate age, mice were weighed at 10:00, food deprived from 18:00 to 10:00, weighed again after night fasting (access to water remained ad libitum) and subjected to OGTT (12–13 mice per group). To perform the OGTT, glucose was administered orally (2 mg/g body weight) after fasting from 18:00 to 10:00, and blood was sampled from the tail vein before and 15, 30, 60, and 120 min after glucose administration. Blood glucose concentrations were determined as described below.

Four days after OGTT, mice of each age group were divided into two subgroups: control and fasting (6–7 mice per subgroup). In the fasting group mice were deprived of food from 18:00 to 10:00 (access to water was ad libitum) and killed by decapitation within a few seconds without anesthesia. Trunk blood was collected in plastic tubes with EDTA and chilled on ice. Plasma was separated by centrifugation and frozen at −20°C until assays. Perigonadal WAT and interscapular BAT were immediately dissected, weighed, and quickly frozen in liquid nitrogen for later measurement of gene expression. Adipose tissues mass indexes were calculated as the ratio of adipose tissue mass to body weight (BW). Samples of thigh muscle were also collected and frozen.

### Biochemical assays

Blood glucose concentrations were determined with a glucometer (One Touch Basic Plus, Lifescan, Russia). Plasma concentrations of glucose, insulin, FFA, and TG were measured using commercial kits (Fluitest GLU, Analyticon Biotechnologies, Lichtenfels, Germany, for glucose; Rat/Mouse ELISA kit, EMD Millipore, Missouri, USA, for insulin; DiaSys Diagnostic Systems GmbH, Holzheim, Germany, for FFA; and DAIKON‐DC, Pushchino, Russia, for TG). Insulin concentrations were not measured in plasma of fasted mice as some samples were lost due to a technical failure (power shortage) beyond our control.

### Relative quantification real‐time PCR

Total RNA was isolated from the WAT, BAT, and muscle tissue samples using TRI Reagent (Ambion) according to the manufacturer's instructions. RNA was used as a template to generate first‐strand cDNA synthesis with Moloney murine leukemia virus (MMLV) reverse transcriptase (Promega, Madison, WI) and oligo (dT) as a primer. For *Slc2a4*, we used the Applied Biosystems TaqMan gene expression assay Mm01245502m1 with *β*‐actin as the endogenous control (TaqMan endogenous controls with FAM dye label and MGB mouse *β*‐actin [ACTB]) and TaqMan Gene Expression Master Mix was used to perform relative quantification by real‐time PCR. For other genes, real‐time PCR was performed with SYBR Green I and ROX dyes. Reactions were performed in 25 *μ*L volume using Master Mix (Syntol, Moscow, Russia), 10 *μ*mol/L of each forward and reverse primers, and 1 *μ*L of cDNA template. The PCR protocol included 40 cycles in total, each cycle with denaturation at 95°C for 15 sec, and annealing and elongation at 60°C for 1 min. The primer sequences were the following: sense 5′‐ caacgagcggttccgatg ‐3′, antisense 5′‐ cactgtgttggcatagagg ‐3′ for *β*‐actin (Gong et al. 2008); sense 5′‐ gggcattcagaggcaaatcag ‐3′, antisense 5′‐ ctgccacacctccagtcattaag ‐3′ for *Ucp1* (Nakamura et al. 2013); sense 5′‐ tcttgatttacacggaggtg ‐3′, antisense 5′‐tcttgtttgtttgtccagtg‐3′ for *Lipe* (Yang and Deeb 1998); sense 5′‐ gactcaccgctgacttcc ‐3′, antisense 5′‐ ctgtctcgttgcgtttgta ‐3′ for *Lipe* (Ying et al. 2013); sense 5′‐ ttcaacactacacgcatccc ‐3′, antisense 5′‐ gccctcatagagccagacc ‐3′; for *Cpt1*b (Kim et al. 2014); and sense 5′‐ tgtttactgacaacttcccc ‐3′, antisense 5′‐ tcatgtatcgggtctttacc ‐3′ for *Ucp3* (Lee et al. 2013). Sequence amplification and fluorescence detection were done with the Applied Biosystems ViiA^™^ 7 Real‐Time PCR System. The reactions were performed in duplicate, and the results were averaged. Relative quantification was performed using the comparative CT method, where CT is the threshold cycle.

### Statistical analysis

All data were expressed as the means ± SEM. Two‐way ANOVA was used to compare all data (excepting blood glucose from OGTT and plasma insulin levels) with age (10, 15, and 30 weeks) and experimental conditions (fed and fasted) as explanatory factors. Blood glucose concentrations during OGTT were analyzed by two‐way repeated measures ANOVA with age (10, 15, and 30 weeks) and time after glucose administration (0, 15, 30, 60, and 120 min) as explanatory factors. Plasma insulin concentrations were measured only in fed animals and were analyzed by one‐way ANOVA with age (10, 15, and 30 weeks) as the explanatory factor. Duncan's multiple‐range test was used for post hoc comparisons between groups. The STATISTICA 6 software package (StatSoft) was used for all analyses. Differences were considered statistically significant at *P* < 0.05.

## Results

### Body weight, tissue weight, and blood parameters

**Figure 1:**
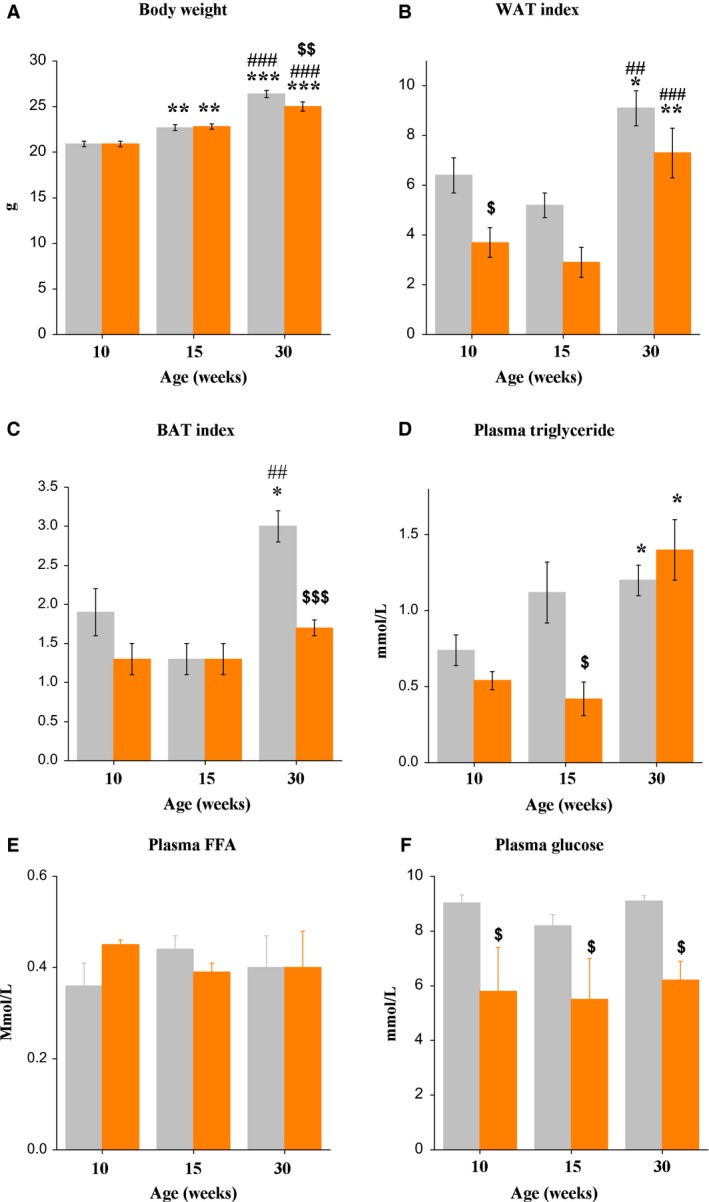
(A) Body weight, (B) perigonadal WAT index, (C) BAT index, (D) plasma triglyceride, (E) free fatty acid, and (F) glucose concentrations in fed (gray bars) and 16 h‐fasted (orange bars) male C57Bl mice of three ages. Data are expressed as the means ± SEM. *P < 0.05, **P < 0.01, ***P < 0.001 versus 10‐week group under the same feeding condition; ^##^ *P* < 0.01, ^###^ *P* < 0.001 versus 15‐week group under the same feeding condition; ^$^ *P* < 0.05, ^$$^ *P* < 0.01, ^$$$^ *P* < 0.001 versus fed mice within the same age group.

Body weight increased with age (*F*_2,65_ = 100.3, *P* < 0.001). Fasting decreased body weight only in 30‐week‐old mice (*P* < 0.01) (Fig. 1A). Paragonadal WAT mass index increased from early to late adult age (*F*_2,36_ = 16.6, *P* < 0.001). Fasting influenced the WAT mass index (*F*_1,36_ = 12.5, *P* < 0.01). The fasting‐induced decrease was statistically significant only in 10‐week‐old mice (Fig. 1B). The interscapular BAT mass index also increased from early to late adult age (*F*_2,36_ = 11.5, *P* < 0.001) (Fig. 1C). Fasting influenced the BAT mass index (*F*_1,36_ = 11.1, *P* < 0.001) and effects were age‐related (*F*_2,36_ = 4.1, *P* < 0.05), with significantly lower BAT mass index in comparison to fed animals only in 30‐week‐old mice (approximately 2.8‐fold) (*P* < 0.001).

**Figure 2:**
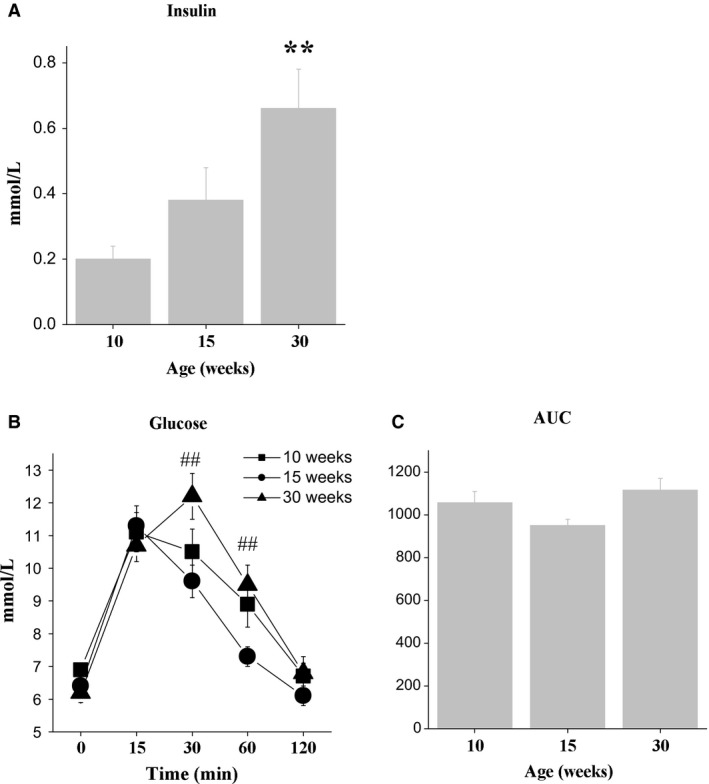
(A) Plasma insulin, (B) blood glucose concentrations from the oral glucose tolerance test (OGTT), and (C) area under the curve (AUC) for glucose in 16 h‐fasted male C57Bl mice of three ages. Data are expressed as the means ± SEM. **P < 0.01 versus 10‐week group; ^#^ *P* < 0.05 versus 15‐week group.

Plasma TG concentrations increased with age (*F*_2,27_ = 7.6, *P* < 0.01) in fed and fasted 30‐week‐old‐mice and were higher than in fed and fasted 10‐week‐old mice (*P* < 0.05 in both cases) (Fig. 1D). Fasting showed a trend toward age‐dependent effects on plasma TG concentrations based on the age × fasting interaction (*F*_2,37_ = 3.0, *P* < 0.07). Fasting significantly decreased plasma TG concentrations (*P* < 0.05) only in early adult mice (Fig. 2A). Age and fasting did not affect plasma FFA concentrations (Fig. 1E). Plasma glucose concentrations in fed mice were unchanged with age. Compared to the fed group, plasma glucose levels were significantly decreased in fasted animals of all ages (*P* < 0.001, in all cases) (Fig. 1D).

Plasma insulin concentrations in fed mice increased from 10 to 30 weeks of age (*F*_2,26_ = 6.6, *P* < 0.05) and were higher in 30‐week‐old mice than in 10‐week‐old mice (*P* < 0.01) (Fig. 2A). Blood glucose concentration dynamics at GTT depended on the age of mice (time × age interaction; *F*_8,170_ = 3.3, *P* < 0.01) (Fig. 2B). In 30‐week‐old mice blood glucose levels were higher than in 15‐week‐old mice at time points 30 and 60 min (*P* < 0.01 in both cases). Age influenced the mean area under the curve (AUC), which represents the index of glucose intolerance (*F*_2,26_ = 3.0, *P* < 0.06, tendency). Moreover in 30‐week‐old mice the mean area under the curve (AUC), which represents the index of glucose intolerance, was somewhat higher than in 15‐week‐old mice (*F*_2,26_ = 3.0, *P* < 0.06, tendency). These data collectively suggest that glucose tolerance was reduced in late adult mice compared to younger mice.

### Skeletal muscle expression of genes related to energy metabolism

**Figure 3:**
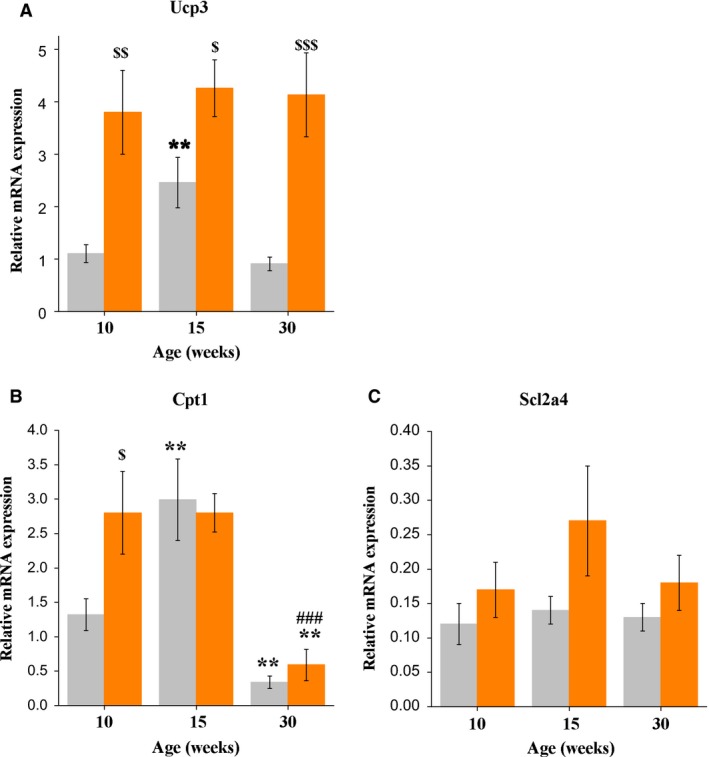
(A) Thigh muscle *Ucp3*, (B) *Cpt*1b, and (C) *Slc2a4* mRNA expression in control (gray bars) and 16 h‐fasted (orange bars) male C57Bl mice of three ages. Data are expressed as the means ± SEM. **P < 0.01 versus 10‐week group under the same feeding condition; ^###^ *P* < 0.001 versus 15‐week group under the same feeding condition. ^$^ *P* < 0.05, ^$$^ *P* < 0.01, ^$$$^ *P* < 0.001 versus fed mice within the same age group. *Ucp*3 – gene of uncoupling protein 3; *Cpt1b –* gene of carnitine palmitoyltransferase 1 b; *Slc2a4* – gene of glucose transporter type 4.

In fed mice, muscle *Ucp3* gene expression changed with age. *Ucp3* mRNA levels in 15‐week‐old mice were higher than in 10‐week‐old mice (*P* < 0.05) and 30‐week‐old mice (*P* < 0.07, tendency). Sixteen hour fasting increased *Ucp3* mRNAs (*F*_1,34_ = 31.4, *P* < 0.001) in all age groups (*P* < 0.01 for 10‐week‐old mice; *P* < 0.05 for 15‐week‐old mice; and *P* < 0.001 for 30‐week‐old mice). After fasting, there were no age‐related differences in muscle *Ucp3* mRNAs (Fig. 3A). In fed mice, muscle *Cpt1* gene expression changed with age in a similar manner (*F*_2,32_ = 21.4, *P* < 0.001). The levels of *Cpt1* mRNAs in 15‐week‐old mice were higher than in 10‐ and 30‐week‐old mice (*P* < 0.01 and *P* < 0.001, respectively). Significantly higher *Cpt1* mRNA levels compared to fed animals were only observed in fasted 10‐week‐old mice (*P* < 0.05) (Fig. 3B). There were no age‐related changes in basal muscle *Slc2a4* mRNA levels; however, fasting increased *Slc2a4* mRNA levels (*F*_1,24_ = 7.0, *P* < 0.05). Fasting‐induced increases in *Slc2a4* mRNA levels were most pronounced in 15‐week‐old mice (*P* < 0.05) (Fig. 3C).

### Adipose tissue expression of genes related to energy metabolism

**Figure 4:**
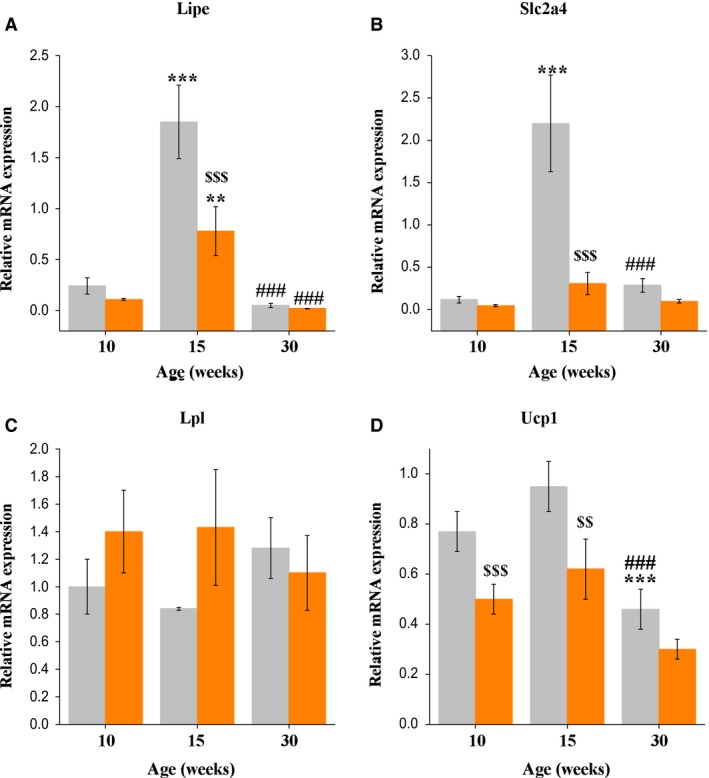
(A) WAT *Lipe*, (B) *Slc2a4*, (C) *Lpl*, and (D) BAT *Ucp1* mRNA levels in fed (gray bars) and 16 h‐fasted (orange bars) male C57Bl mice of three ages. Data are expressed as the means ± SEM. *^*^ *P* < 0.01, **^*^ *P* < 0.001 versus 10‐week group under the same feeding condition; ^###^ *P* < 0.001 versus 15‐week group under the same feeding condition; ^$$^ *P* < 0.01, ^$$$^ *P* < 0.001 (Duncan's post hoc test). *Lpl –* gene of lipoprotein lipase; *Lipe –* gene of hormone‐sensitive lipase; *Slc2a4 –* gene of glucose transporter type 4; *Ucp1* – gene of uncoupling protein 1.

In fed mice, WAT mRNA expression of *Lipe* changed with age (*F*_2,28_ = 37.8, *P* < 0.001). *Lipe* mRNAs were elevated in 15‐week‐old mice compared to 10‐ and 30‐week‐old mice (*P* < 0.001 in both cases). Fasting decreased WAT *Lipe* mRNA levels (*F*_1,28_ = 9.9, *P* < 0.01). The effect depended on the age of the mice (significant interaction of age × experimental group: *F*_2,28_ = 6.2, *P* < 0.01). Fasting decreased *Lipe* mRNA levels only in 15‐week‐old mice (*P* < 0.001) (Fig. 4A). In fed mice, WAT mRNA expression of *Slc2a4* changed with age in a similar manner (*F*_2,27_ = 16.9, *P* < 0.001). *Slc2a4* mRNAs were elevated in 15‐week‐old mice compared to 10‐ and 30‐week‐old mice (*P* < 0.001, in both cases). Fasting decreased WAT *Slc2a4* mRNA levels (*F*_1,27_ = 15.9, *P* < 0.001). The effect was age‐dependent (significant interaction of age x experimental group: *F*_2,27_ = 10.5, *P* < 0.001). Fasting decreased *Slc2a4* mRNA levels only in 15‐week‐old mice (*P* < 0.001) (Fig. 4B). Age and fasting did not affect WAT *Lpl* gene expression (Fig. 4C). In fed mice, BAT *Ucp1* gene expression changed with age (*F*_2,33_ = 14.1, *P* < 0.001), it was lower in 30‐week‐old mice than in 10‐ and 15‐week‐old mice (*P* < 0.05 and *P* < 0.001, respectively). Fasting decreased BAT *Ucp1* mRNA levels (*F*_1,32_ = 14.8, *P* < 0.001). Fasting‐induced decrease in the *Ucp1* mRNA levels was most pronounced in 10‐ and 15‐week‐old mice (*P* < 0.001 and *P* < 0.01, respectively) (Fig. 4D).

## Discussion

Researchers have accumulated considerable data showing that during normal development in humans (Daviglus et al. 2003; Mizuno et al. 2004; Yoneshiro et al. 2011) and rodents (Gruenewald and Matsumoto 1991; Jacobson 2002; Sasaki 2015), middle aged individuals (defined as 40–50 years in humans [Mizuno et al. 2004] and 9–12 months in mice [Flurkey et al. 2007; Gruenewald and Matsumoto 1991]) exhibit higher incidence of obesity and impaired glucose metabolism compared to younger ages. Our study of metabolic indexes in mice from three age groups, including young (10 weeks), early adult (15 weeks) and late adult (30 weeks) (Flurkey et al. 2007) revealed that age‐related changes in carbohydrate‐lipid metabolism occur in mice long before middle age, that is, in the late adult stage. During normal development, late adult mice show evidence of the onset of obesity, including a twofold increase in WAT and BAT indexes, increased plasma triglyceride and insulin levels, and decreased glucose tolerance. The mechanisms causing changes in energy metabolism at late adult age have yet to be identified.

The results obtained in this study revealed that in fed mice, mRNA levels for genes controlling FFA oxidation in muscles (*Ucp3*,* Cpt1*) and lipolysis (*Lipe*) and glucose uptake (*Slc2a4*) in WAT showed considerable age‐related changes: that is, increased from young to early adult age and decreased from early to late adult age. Similar age‐related changes in gene expression in muscles and WAT in mice have not yet been reported, and the mechanisms are unknown. Increased expression of these genes in muscles and WAT of early adult mice compared to young mice is likely caused by age‐related activation of several hormone systems, especially androgenic testis function. In male C57Bl mice, there was a significant increase in plasma androgen level from 10 to 15 weeks of age (Osadchuk et al. 2016). Androgens are known to activate the growth hormone (GH)/insulin‐like growth factor (IGF1) axis (Cummings and Merriam 1999). Together androgens and the GH/IGF1 axis hormones activate anabolic pathways (Yakar and Isaksson 2016) and energy substrate metabolism (Davidson 1987; Kelly and Jones 2013; Richelsen et al. 2000; Varlamov et al. 2015). However, an age‐related decrease in gene expression in muscles and WAT cannot be caused by androgenic activity as blood testosterone levels and testicular production in late adult mice remains as high as in early adult mice (Osadchuk et al. 2016); thus, these factors will require additional study.

Interestingly, enhancement and subsequent decrease in expression of the study genes in muscles and WAT completely correspond to age dynamics of voluntary physical activity described for C57Bl mice (Figueiredo et al. 2009). According to Figueiredo et al., voluntary physical activity levels peak at 14–15 weeks of age and then decline during the subsequent 10 weeks, reaching a new plateau of activity at approximately 25–30 weeks of age. Correlation between physical activity, *Cpt1*, and *Ucp3* gene expression in muscles as well as intensity level of lipolysis processes in WAT was shown in several studies. In rats and C57Bl mice, physical activity increases *Cpt1* mRNA and protein expression (Niu et al. 2010; Shen et al. 2015) and *Ucp3* mRNA and protein expression (Tsuboyama‐Kasaoka et al. 1998; Watt et al. 2004); in WAT, it enhances lipolysis and the level of phosphorylated HSL (Higa et al. 2014; Ogasawara et al. 2010). Apparently, up‐regulation of gene expression in muscle (*Ucp3*,* Cpt1*) and WAT (*Lipe Slc2a4*) in early adult mice was an adaptive response that aimed to increase energy production in the period of intensive growth, reproductive and physical activity. Down‐regulation of gene expression in late adult mice contributed to decreased FFA oxidation and induction of fat storage in WAT and BAT. The decrease in BAT mRNA levels of *Ucp1* (molecular marker of energy expenditure for thermogenesis) detected in late adult mice (as compared to younger mice) could also contribute to obesity (Keipert et al. 2014).

Our study revealed that fed late adult mice concurrently with starting obesity demonstrated impaired glucose metabolism including increased plasma insulin levels, decreased glucose tolerance compared to younger mice, and decreased expression of *Slc2a4* gene in WAT (compared to early adult mice). Glut4 is an insulin‐dependent glucose transporter whose activity influences glucose uptake at the level of the whole organism. It was shown that age‐related reduction in WAT *Slc2a4* gene expression is associated with the development of decreased glucose tolerance (Li et al. 2014) and insulin resistance (Hofmann et al. 1991), while *Slc2a4* overexpression improves insulin resistance (Carvalho et al. 2005). It can be assumed that in the late adult stage, glucose tolerance in mice was also reduced due to an increase in the proportion of WAT because adipocyte products are known to suppress intracellular insulin signaling pathways (Ye 2013). Data on impaired glucose metabolism at late adult ages are in agreement with the results of other studies demonstrating increased insulin and blood glucose concentrations (Stenbit et al. 1997) and decreased sensitivity to insulin in 30‐week‐old C57Bl mice (Mizuno et al. 2004).

The age‐related decrease in *Ucp3* gene expression in muscles and *Slc2a4* in WAT observed in fed late adult mice had no effect on the transcriptional responses of these genes to fasting compared to response in other age groups. During fasting, *Ucp3* gene expression in mice sharply increased in all ages, which corresponds to data obtained in other studies with rodents (De Lange et al. 2006; Sánchez et al. 2009) and humans (Millet et al. 1997). *Ucp3* gene expression activation is an important component of adaptation to fasting since UCP3 lowers mitochondrial membrane potential, protects muscle cells against an overload of fatty acids, and reduces excessive production of reactive oxygen species (Amat et al. 2007). Fasting is known to cause not only metabolic system load but also emotional stress and also increases glucocorticoid levels in peripheral blood (Bazhan et al. 2017; Viscarra and Ortiz 2013) stimulating *Ucp3* gene expression in mice (Amat et al. 2007; Nagase et al. 2001). It seems that the response to fasting‐induced stress in mice at the studied ages did not differ, resulting in similar increases in *Ucp3* gene expression in muscles.

Transcriptional responses to fasting for the *Cpt1* gene in skeletal muscle in late adult mice differed considerably from that of younger mice. Young mice in response to fasting significantly increased muscle *Cpt1* gene expression. This result is in accordance with findings from other studies of rat muscles (De Lange et al. 2006). For early and late adult mice, fasting failed to change *Cpt1* mRNA levels. We believe that in early adult mice the absence of stimulation can be explained by the “ceiling” effect, i.e., *Cpt1* mRNA level in fed mice was as high as in young fasted mice. Fasting did not increase muscle Cpt1 mRNA level in early adult mice because the gene transcription was already at, or near, a pinnacle point, due to activation induced by some age‐related factors. The absence of change in late adult mice is believed to represent age‐related impairment of reaction to a specific factor that is activated during fasting, probably to FFA. Conservation of low levels of muscle *Cpt1* gene expression during fasting could prevent adaptation due to the use of fatty acids as an energy source in muscles.

Adult C57Bl mice are widely used to study different aspects of carbohydrate‐lipid metabolism. It should be noted that considerable age‐related changes in gene expression in muscles as well as WAT and BAT at postpubertal stages precedes the late adult age and that the onset of obesity at late adult age can influence these results.

Thus, this study for the first time revealed that during normal development even at late adult age expression of genes involved in TG hydrolysis, WAT glucose uptake, muscle FFA oxidation, and BAT thermogenesis sharply decreased (compared to early adult ages). However, the conclusion of the role of enzymes of peripheral metabolic organs in impaired lipid and glucose metabolism at late adult age can only be drawn after measuring expression of the respective proteins and enzyme activity. The study of mechanisms triggering obesity during normal development in mice is relevant because obesity at a later age predisposes them to life‐threatening conditions such as insulin resistance, type 2 diabetes, and cardiovascular disease.

## Conflict of Interest

None declared.
